# Factors associated with seeking treatment for postpartum morbidities in rural India

**DOI:** 10.4178/epih/e2014026

**Published:** 2014-10-30

**Authors:** Aditya Singh, Abhishek Kumar

**Affiliations:** 1 Global Health and Social Care Unit, School of Health Sciences and Social Work, University of Portsmouth, Portsmouth, UK; 2 International Institute for Population Sciences, Mumbai, Maharashtra, India

**Keywords:** Postpartum morbidity, Healthcare utilization, Rural India, District Level Household Survey-3, Treatment-seeking

## Abstract

**OBJECTIVES:**

To understand the prevalence of postpartum morbidities and factors associated with treatment-seeking behaviour among currently married women aged 15-49 residing in rural India.

**METHODS:**

We used data from the nationally representative District Level Household Survey from 2007-2008. Cross-tabulation was used to understand the differentials for the prevalence of postpartum morbidities and treatment-seeking behaviours across selected background characteristics. Two-level binary logistic regression was applied to understand the factors associated with treatment-seeking behaviour.

**RESULTS:**

Approximately 39.8% of rural women suffered from at least one of the six postpartum morbidities including high fever, lower abdominal pain, foul-smelling vaginal discharge, excessive bleeding, convulsions, and severe headache. Morbidities were more prevalent among poor, illiterate, Muslim, and high-parity women. About 55.1% of these rural women sought treatment/consultation for their problems. The odds of seeking treatment/consultation increased as economic status and years of schooling among both the woman and her husband increased. Poor, uneducated, unemployed, Hindu, and tribal women were less likely to seek treatment/consultation for postpartum morbidities than their counterparts were. The odds of seeking treatment/consultation decreased as the distance to the nearest private health facility increased. Most women visited a private hospital (46.3%) or a friend/family member’s home (20.8%) for treatment/consultation. Only a small percentage visited publicly funded health institutions such as a primary health centre (8.8%), community health centre (6.5%), health sub-centre (2.8%), or district hospital (13.1%). Rural women from the northeast region of India were 50% less likely to seek treatment/consultation than women from the central region were.

**CONCLUSIONS:**

Providing antenatal and delivery care, and ensuring nearby government healthcare facilities are available to serve rural women might increase the likelihood of care-seeking for postpartum morbidities. Targeted interventions for vulnerable groups should be considered in future policies to increase the likelihood women will seek treatment or advice postpartum.

## INTRODUCTION

The postpartum period, which begins one hour after the delivery of the placenta and continues until 42 days after birth, is a critical transitional period for the health of a woman [1]. The importance of this period lies in the fact that approximately 61% of all maternal deaths occur in this period as compared to 24% during pregnancy and 16% during delivery. During this period, women also suffer from a number of obstetric morbidities such as haemorrhage, pregnancy-related hypertension, pulmonary embolism, and puerperal sepsis etcetera [2]. Although not all postpartum morbidities lead to maternal mortality, a few select ones contribute heavily to global mortality, and the disease and disability burden. Postpartum haemorrhage and hypertension, for example, cause 27.1% and 14.0% of all maternal deaths worldwide, respectively [3].

For every maternal death, at least another 30 women suffer serious illnesses or debilitating injuries. Thus, maternal mortality is just the tip of the iceberg and maternal morbidity is the base [4]. Although there is no systematic data available for geographical distribution, it is estimated that the burden of maternal morbidity is highest in developing countries where the postpartum period tends to be most neglected [5]. While the World Health Organization guidelines underline the importance of follow-up examinations during the postpartum period from an experienced, trained healthcare professional to prevent maternal morbidity and subsequent mortality [6], there prevails a general indifference to postpartum care in most developing countries, which is not only reflected in government policy documents but also in the literature, where research on effective interventions is scarce [1].

Unfortunately, the situation in India is not encouraging either [7]. Most of the programs launched by the government of India in past decades have focused on reduction in maternal mortality while maternal morbidity continues to remain a neglected area despite the fact it not only adversely affects women’s physical, mental and sexual health but also may have serious implications for social and economic wellness, self-esteem and body image [4]. Thus, the focus has been mainly on antenatal care, skilled birth attendance, and institutional delivery. Consequently, no specific program focusing on postpartum care exists in India [8]. Not surprisingly, the level of postpartum care in India is one of the lowest among developing countries, and women frequently fail to seek medical care and consultation from healthcare providers. As a result, the burden of postpartum morbidities in India is as high as many other developing countries. To establish program priorities and design appropriate intervention strategies for reducing the burden of postpartum morbidities by enhancing coverage of healthcare service utilization, it is imperative to have a clear understanding of the factors that influence treatment-seeking for postpartum morbidities in rural India.

A review of the existing literature on this issue indicates that most previous studies in India have been performed at the community level and focus on overall maternal/obstetric morbidity rather than postpartum morbidity specifically [9-13]. The findings of these previous studies cannot be easily generalised. To our knowledge, no national level study has been conducted on this issue. Scarcity of nationally representative data could be one of the reasons. Even the third wave of the National Family Health Survey, conducted during 2005-2006, included data on only two postpartum morbidities. However, the District Level Household Survey conducted during 2007-2008 has collected information on six postpartum morbidities and has asked a few questions regarding the treatment-seeking behaviour among women as well [14]. Therefore, using this dataset, the present study aims to examine the prevalence of selected postpartum morbidities and factors associated with treatment-seeking for these morbidities among currently married women aged 15-49 years in rural India.

## MATERIALS AND METHODS

### Data

Data from the third round of the District Level Household Survey, a nationally representative survey conducted in India during 2007-2008, were used. This survey is one of largest demographic surveys ever conducted in India. The basic aim of the survey was to provide reliable estimations of maternal and child health, family planning, and other reproductive health indicators at district level. Information about sampling procedures can be obtained from the survey’s website and published reports [15]. The survey collected data on 174,913 currently married women aged between 15-49 years who lived in rural India and had delivered at least once during the previous three years at the time of the survey.

### Dependent variable

Data on the participants’ most recent birth in the previous three years were collected by asking whether they had experienced any of the following health problems during the first six weeks after delivery: high fever, lower abdominal pain, foul-smelling vaginal discharge, excessive bleeding, convulsions, and/or a severe headache. Women who reported any of these health problems were further asked if they sought consultation or treatment for their health problems, and answers were collected as yes or no. This binary variable was used as the dependent variable in our analysis and was categorized as treatment not sought (0) or treatment sought (1). The treatment-seeking question was asked for any reported morbidities together in one question. In the present study, the phrase ‘treatment seeking’ includes seeking a consultation and/or treatment for one of the postpartum morbidities. Therefore, the results should be interpreted with caution.

### Independent variables

Health service utilization is a complex behavioural phenomenon. Previous studies have found that health services utilization is related to the availability, quality, and cost of services as well as the social structure, health beliefs, and personal characteristics of the users. Several models of maternal healthcare utilization have been proposed, including the behavioural model of health services utilization [16], health belief model [17], and three delays model [18]. According to the behavioural model, an individual’s access to and use of health services is considered a function of three characteristics: (i) predisposing factors, the socio-cultural characteristics of the individuals prior to their incident illness, such as education, occupation, ethnicity, social networks, social interactions, culture, attitudes, values, knowledge of the health care system, age, and gender; (ii) enabling factors, the logistical aspects of obtaining care, including the means and knowledge of how to access health services, income, existing health insurance, a regular source of care, ability to travel, extent and quality of social relationships, available healthcare personnel and facilities, and the required waiting time at these facilities; and (iii) need factors, the factors that require immediate health service utilization, including perceived and evaluated needs. Perceived needs are used to understand the reasons for seeking care and adhering to a medical regimen, while evaluated needs are used to evaluate the kind and amount of treatment provided after a patient has presented to a medical care facility [16].

We have included a number of socioeconomic variables such as religion, caste, place of residence, education, employment status, and economic status in the analysis. These variables have been found to have associations with the healthcare-seeking behaviour of mothers in different settings across the world. The factors such as caste, religion, and education are used to represent the social identity of each respondent. A household’s economic status is measured using the wealth index. The mother’s employment captures her ability to earn an income that she could potentially use for accessing healthcare services. The effects of a person’s social identity on her health services utilization may be indirect; however, we have included some factors that provide direct measures of a woman’s ability to access healthcare, such as her healthcare utilization history during pregnancy and delivery, geographical proximity to healthcare facilities, accessibility of the village by an all-weather road, and level of village development. In low-resource settings, married women are often reluctant to seek medical treatment because of a lack of privacy and female doctors, the cost of treatment, and their inferior social status. In rural communities, cultural beliefs and practices may also hinder the health-seeking behaviours. Appendix 1 lists all independent variables used in our analysis.

### Statistical analysis

A contingency table was used to understand differences between the prevalence of postpartum morbidities and seeking treatment across selected background characteristics. To understand the association between treatment-seeking behaviour and independent variables, two-level binary logistic regression was used. We preferred multi-level regression over single level regression because the former is able to take into account the hierarchical nature of the dataset used in this study [19]. A two-level logit model can be written as follows:

logitπij=logπij1-πij=β0+β1xij+β2yij+β3zj...+μj

where *i* and *j* are the level one (individual) and level two (primary sampling unit, village, or community) units; π_ij_ is the likelihood one sought treatment or consultation for the *i*th women in the *j*th village (primary sampling unit); the β variables are fixed coefficients; μ_j_-N (0, σ^2^_j_) represents the random effects for the *j*th village (primary sampling unit); and x, y, and z are the individual, household, and village characteristics, respectively.

We fitted the two-level null model using the second order penalized quasi-likelihood estimation method and found that approximately 18% of the total variation in seeking treatment existed at the community level. The Wald test was carried out to test the significance of the residuals at the village level, and these village/community residuals were statistically significant, thus suggesting the need for a two-level model. In addition, evidence of multicollinearity using the variance inflation factor was tested for as a post-estimation procedure. The small variance inflation factor (2.43) indicated the absence of any significant collinearity among the independent variables. The results are presented in the form of odds ratios (OR) with 95% confidence intervals.

## RESULTS

### Prevalence of postpartum morbidities

**Figure 1.**
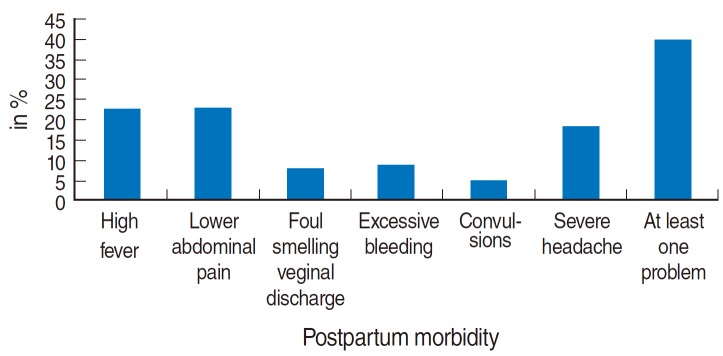
Prevalence of postpartum morbidities among mothers (aged 15-49 years) in rural India, 2007-2008.

Table 1 shows the prevalence of each of postpartum morbidities among our population of currently married women in rural India during 2007-2008. The prevalence of high fever, lower abdominal pain and severe headache was 22.6, 22.9, and 18.4%, respectively. Severe bleeding and foul-smelling vaginal discharge was prevalent among 8.8 and 7.8% women, respectively. Convulsions were reported by only 4.9% of women. In all, approximately 39.8% of these rural women reported at least one of the six postpartum morbidities discussed above (Figure 1).

We examined the bivariate differentials to explore how the prevalence of different postpartum morbidities varied across selected socioeconomic and demographic characteristics (Table 1). The prevalence of having one or more morbidity was highest among the poorest mothers (44.8%) and lowest among the wealthiest mothers (29.1%). The morbidity burden among the poorest mothers was more than double that of the wealthiest mothers. For instance, the prevalence of convulsions was only 2.6% vs. 5.7% among the wealthiest and poorest, respectively. In addition, a progressive decline in the prevalence of morbidities was observed with rising education levels of the woman and her husband. The prevalence of one or more morbidities was highest among illiterate women (43.3%) and lowest among women with at least secondary education (26.6%). This discrepancy between the mother’s education levels also persisted for individual morbidities. The burden of postpartum morbidity increased with the parity (total number of births) of each woman. For instance, the prevalence of having one or more morbidities among mothers with a very low parity (birthing one child) was 36.9% vs. 48.2% among mothers with high parity (birthing six or more children)

Morbidity varied little across age and employment categories. However, the prevalence of having one or more morbidity did vary across religious and caste groups. Muslim mothers were disproportionally burdened (50.0%) compared to the Hindu mothers (39.7%) and mothers of other religions (28.1%). A similar pattern was found for each of the six postpartum morbidities. Among the different caste groups, the prevalence of having one or more morbidities was lower among scheduled tribes (ST) than that among scheduled castes (SC, 41.6%), other backward castes (OBC, 41.8%), and the forward castes (FC, 40.5%) were. The prevalence of having one or more morbidity was lowest among mothers who received their first antenatal care (ANC) visit in the first trimester (31.5%), while it was highest among those who had less than four ANC visits and received their first ANC in the second or third trimester (43.5%). Mothers delivering at home (42.4%) were more likely to have one or more morbidity than those who delivered at a healthcare facility (35.8%) were. The prevalence of morbidities among mothers who experienced delivery complications (49.3%) was almost twice the prevalence among mothers with no delivery complications (23.7%). The burden of morbidities among mothers living in south India was lowest (24.5%), yet that in the east region of India was the highest (50.3%). A similar pattern was found for each of the six postpartum morbidities.

### Differentials in treatment-seeking

Table 2 reports the proportions of women who sought treatment for postpartum morbidities by their background characteristics and the community-level variables. Overall, approximately 55.1% of women sought treatment for postpartum morbidities in rural India. Approximately 70% of women from the wealthiest quintile sought treatment vs. only 48% who sought treatment from the poorest quintile. Only 51% of those mothers with no schooling sought treatment, while 71% of mothers with ten or more years of schooling sought treatment. A similar increased trend in the proportion of women seeking treatment for postpartum morbidities was witnessed as the husband’s education increased. In terms of their religious affiliations, only 45% women from religions other than Hindu and Muslim sought treatment versus the 55% and 60% of Hindu and Muslim women who sought treatment, respectively.

The proportion of treatment-seeking women was substantially lower (51%) among ST women than that among OBC women (58%) and FC women (60%) were. Among agricultural workers, approximately 53% sought treatment as opposed to 62% of the professional/service/production workers who sought treatment. The proportion of women who sought treatment for postpartum morbidities was higher among those who had their first ANC in the first trimester and had received at least four total ANCs (72%) than that among those who never received an ANC (42%) was. Similarly, the proportion of women who sought treatment among those who delivered in a healthcare facility was greater (66%) than that among those who delivered at home (49%) was.

With increasing distance between a healthcare facility and a village, a progressive decrease in the proportion of women seeking treatment was observed. For instance, approximately 60% of women whose village was within one kilometre of a government/private healthcare facility sought treatment. However, only approximately 53% of women living in villages within 5 km from a healthcare facility sought treatment. In addition, a progressive increase in the proportion of women seeking treatment was observed with an increased level of village development. There was also a marked regional variation in the treatment-seeking behaviour; the proportion of women seeking treatment ranged from 76% in the southern region to 42% in the northeast region of India.

### Factors associated with treatment-seeking behaviour

Table 3 presents the OR for seeking treatment due to postpartum morbidities. The total number of participants included in the final regression model was reduced to 65,706 women due to missing values for many variables. All unadjusted ORs were highly significant. However, in the final model, the magnitude of the ORs was significantly reduced and almost all response variables were statistically significant. Most of the variables were found to be significant and in the expected direction. At the community level, only region and distance to a private healthcare facility were found to be statistically significant.

There was a progressive increase in the ORs for seeking treatment from the lowest to the highest wealth quintiles. For instance, women from the second, third, and fourth wealth quintiles were 8, 10, and 23%, respectively, more likely to seek treatment than women from the lowest quintiles were. Women who had 10% or more years of schooling were approximately 20% more likely to seek treatment for their postpartum morbidities than were those who never went to school. Similarly, there was a progressive increase in the ORs of seeking treatment across the number of years their husbands reportedly attended school. The parity of each mother was also strongly associated to seeking treatment. Mothers with a high parity (six or more children) were 34% more likely to seek treatment than those with a low parity were (one child). In addition, Muslim women were 30% more likely to seek treatment than the Hindu women were.

Caste was also significantly associated with seeking treatment. Women from the SC, OBCs, and FC were 58, 62, and 52% more likely to seek treatment regarding their postpartum morbidities than ST women were, respectively. Moreover, women who worked in a professional/service/production job were 17% more likely to seek treatment than the unemployed were. Women who had four or more ANC visits in the first trimester were two times more likely to seek treatment than women who had never received an ANC were. Similar results were found among women who had more than four ANC visits in the second or third trimester. Women who had their delivery at a healthcare facility were approximately 60% more likely to seek treatment than those who delivered at home without any assistance from a skilled health professional were. In addition, women living in villages more than 5 km away from a healthcare facility were slightly less likely (7%) to seek treatment than women living in the villages close to a health facility (less than 1 km away) were. The ORs for seeking treatment were 49 and 32% higher for women living in the southern and the western regions than the ORs among those living in the central region were, respectively. Conversely, the ORs among women living in the northeast region were approximately 50% lower than that among those living in the central region were.

### Type of health facilities visited for treatment/consultation

**Figure 2.**
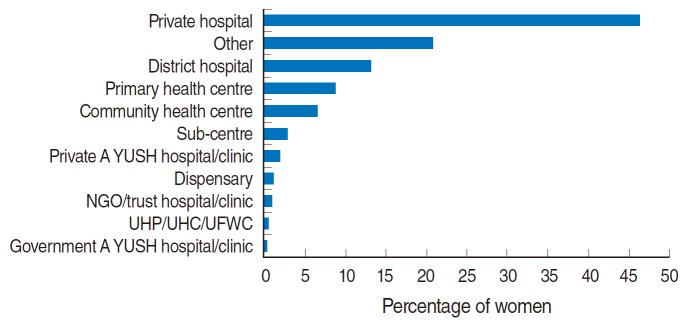
Percent distribution of the place where consultations or treatments for postpartum morbidities were sought, rural India, 2007-2008. NGO, Non-governmental organization; UHP, Urban health post; UHC, Urban health centre; UFWC, Urban family welfare centre.

The District Level Household Survey asked participants to describe the type of treatment/consultation facility that they had visited. Approximately 46.3% women reported that they had visited a private hospital for treatment/consultation for their postpartum morbidities. The proportions of women who visited a district hospital, primary healthcare centre, community health centre, or health sub-centre were 13.1, 8.8, 6.5, and 2.8%, respectively (Figure 2).

## DISCUSSION

Using data from the third round of the District Level Household Survey, this study examined the relationship between seeking consultation/treatment for postpartum morbidities among women in rural India. Our study adds to prior research in three important ways. Firstly, to our knowledge, this national-level study is the first to examine the prevalence of postpartum morbidities and the factors affecting treatment or advice-seeking for postpartum morbidities among rural women. Secondly, in addition to individual level factors, we also examined the effects of community-level factors such as the accessibility of the village by an all-weather road, the distance and availability of private and public healthcare facilities, and the level of village development, among others. All of these factors might influence the treatment-seeking behaviour of women, but have not been taken into consideration in previous studies. Thirdly, unlike previous studies, this study uses a two-level regression analysis that estimates standard errors to a higher degree of accuracy than single-level regressions do and leaves little possibility of underestimating the significance of the variables in question.

The results revealed that approximately two-fifths of our total population suffered from at least one of the six postpartum morbidities, which were high fever, lower abdominal pain, foul-smelling vaginal discharge, excessive bleeding, convulsions, and severe headache. Large socioeconomic differences among the prevalence of having one or more morbidity were found. Morbidity among the poorest mothers was more than double that among the wealthiest mothers. Muslim and high-parity women were also disproportionally burdened by postpartum morbidities. Approximately half of these women reported seeking treatment for one or more morbidities.

In our regression analysis, multiple factors at both the individual- and community-level remain statistically significant until the final model. In addition, the wealth index, a proxy of each household’s economic status, was positively associated with the treatment-seeking behaviour of these women. Women from wealthy households tend to be more educated and have more autonomy than women from poor households do. Moreover, wealthier women are also more likely to be able to afford healthcare than are poorer women whose earnings are typically spent on daily living expenses, like food, leaving behind little or no available funds to spend on healthcare [20].

Receiving a formal education has been found to be an effective means of achieving autonomy in one’s family and gaining employment, thereby achieving more economic independence than women without a formal education typically have [21]. This also encourages exposure to things outside of the household/village, which might help to further educate these women pregnancy, the related complications, how to receive care, the life-threatening and high-risk of potential postpartum complications, and the related care postpartum. Moreover, educated women tend to be confident as well as report have feelings of self-worth and self-confidence. These women have also been found to be able to communicate with their husbands and other family members on health-related issues [22]. A husband’s education level not only decides the financial resources available in the household but also the level of his understanding about the healthcare needs of his wife. In traditional societies like rural India, many restrictions are placed on a woman’s freedom. Therefore, a husband’s attitude and knowledge about his wife’s health likely plays an important role in determining her treatment-seeking behaviour.

Analysis of the predictors of seeking treatment for postpartum morbidities confirmed some expected patterns, but also yielded some surprising results. The common view is that Muslim women are less likely than Hindu women are to make use of medical services, but in this study, the reverse is true. We found that Muslim women were more likely to seek treatment for postpartum morbidities than Hindu women were. This interesting result should be investigated further as it may have important implications on programs that aim to increase access to postpartum care for Hindu women. These results have also been confirmed by those of two previous studies [23,24].

Castes seem to have a profound impact on the treatment-seeking behaviour among women in rural India. Women from the SC, OBC, and FC groups were more likely to seek treatment postpartum than women in the ST were. This finding is not surprising given that ST are socio-economically disadvantaged indigenous groups living in the mountains, dense forests, and typically inaccessible villages, where healthcare providers and facilities are sparse [25]. Furthermore, the existing healthcare facilities in these secluded areas are crippled by a lack of accessibility, poor infrastructure, large-scale absenteeism, a shortage of human resources, and poorly trained, unmotivated manpower. However, higher castes have a high educational level and economic status, and they participate in community development activities; therefore, these individuals tend to command considerable influence in society and have easy access to medical and healthcare facilities.

Working outside the home not only makes women financially strong but also provides the opportunity to be involved in making decisions about the family. Exposure to the world outside of their home provides opportunities to become educated on health and healthcare-related issues, which might provide the confidence to seek treatment/consultation from healthcare professionals and facilities [24]. The results of our study indicate that women who have worked in professional/service/production jobs, as farmers, or as agricultural labourers are more likely to seek treatment than are those who are unemployed. However, the relationship is slightly weaker among women who have worked as labourers and farmers.

Ensuring that an adequate number of ANCs and skilled health professional-assisted deliveries are provided for women in rural India could not only substantially reduce the likelihood of postpartum morbidities but also increase the likelihood a women would seek treatment or consultation in case a postpartum morbidity occurs [23]. Increasing the number of ANCs would also provide healthcare providers with an opportunity to educate mothers about postpartum complications, related treatments, and locations of healthcare facilities she can visit to seek further care. Similarly, giving birth in a healthcare facility or in the presence of a skilled healthcare professional also provides an opportunity to interact with the mother and advise her about the potential postpartum morbidities that can result.

Although, individual factors remain important predictors of whether women will seek treatment, the distance to the nearest private healthcare facility was a statistically significant community-level predictor in our analysis. The odds of seeking treatment decreased with increased distance to a healthcare facility. In rural India, the government healthcare system is still very poor. Healthcare facilities do not have enough human resources or basic facilities with electricity, water, equipment, or vehicles [14]. The struggling government healthcare system in rural areas also has a poor public image and huge shortages of nurses and doctors are still common in the many states, despite several efforts to increase the number of health workers under the National Rural Health Mission [26]. Decades of neglect of the public healthcare system has given way to the dominance of private healthcare providers in rural areas. Moreover, the majority of private practitioners (mostly males) in rural India also have no formal degree or training in maternity care.

The higher ORs for seeking treatment found among women from the southern and western regions may be attributed to their good socio-economic and demographic conditions and relatively superior public healthcare systems. On the contrary, the low ORs among women living in the northeast region may be attributed to their inability to access care, their low socio-economic status, and dysfunctional public healthcare system [22]. Private practitioners in these states dominate the healthcare scene and not only charge high consultation and prescription fees but also charge for medicine [27]. Therefore, a large majority of rural women, especially those who cannot afford private health care, forego seeking consultation or treatment.

Previous studies have noted that government-run reproductive and child health programs in urban and rural areas of India have consistently focused on improving the coverage of antenatal and delivery care services [28]. It is unclear why postpartum care has not been included as part of the continuum of maternity care in the public healthcare policy in India since a great majority of maternal deaths occur during the postpartum period and providing proper care could reduce postpartum mortality substantially. Recent efforts to improve maternal health in India have also not paid attention to the importance of postpartum care. For instance, a conditional cash-transfer scheme known as the Janani Suraksha Yojana (Mother Protection Scheme) under the National Rural Health Mission was started to increase institutional deliveries in rural areas, however has not made postpartum follow-ups mandatory for government healthcare workers. Mass media messages can increase awareness about postpartum care; however, no public announcements such as on the television or radio related to postpartum care exist. When postpartum care is given least priority among healthcare providers, neither health workers nor new mothers try to seek advice and treatment for postpartum morbidities, which frequently result.

This study has few limitations. Firstly, other variables of interest such as the autonomy among these women, local practices, and traditional customs related to maternity complications in the community, the role of family members (especially the mother-in-law), the quality of services, and others that have been found to be associated with the utilization of maternity services were not measured during the survey period. Secondly, some variables such as the employment status of the mother, wealth index, and village development index represent the conditions at the time of the interview, not at the time when the child was born. Finally, we analysed only those postpartum complications that were included in the survey questionnaire.

In conclusion, the postpartum morbidity is an important but complex public health problem in rural India. The prevalence of postpartum morbidities is higher among poor, illiterate, Muslim, and high-parity women. Some vulnerable groups of women (poorest, uneducated, unemployed, Hindu, and tribal mothers) are less likely to seek treatment/consultation for postpartum morbidities. Future policies should aim to increase the level of awareness about postpartum morbidities and their related treatments, especially among women belonging to these groups. Our findings suggest that efforts should be made to increase the uptake of antenatal and delivery care as well as increase the physical accessibility to healthcare facilities. In addition, mothers with low parity should also be included in the target population since they are also not likely to seek treatment/consultation. Almost half of women in this study sought treatment from private hospitals. This may have serious implications for the poor in rural India. Therefore, future studies can explore the factors that influence rural women’s decision to seek care from private providers.

## Figures and Tables

**Table 1.** Prevalence of postpartum morbidities by selected background characteristics among currently married women (aged 15-49 years) in rural India, 2007-2008

Socio-economic characteristics	High fever	Lower abdominal pain	Foul-smelling vaginal discharge	Excessive bleeding	Convulsions	Severe headache	At least one morbidity	No. of women^1^
Wealth index								
Poorest	28.3	26.1	9.5	9.4	5.7	21.2	44.8	43,239
Poor	25.9	25.5	8.8	9.5	5.9	21.0	43.6	43,204
Middle	21.4	22.7	7.6	9.0	4.9	18.4	39.2	38,942
Wealthy	17.2	19.3	6.1	8.1	3.8	15.2	34.5	32,362
Wealthiest	13.2	15.4	4.3	6.9	2.6	11.2	29.1	17,132
Mother’s education								
No education	27.0	25.0	8.8	9.0	5.4	20.8	43.3	87,728
Primary school	22.1	23.6	8.0	9.2	5.1	18.8	40.0	27,020
Secondary school	17.0	19.9	6.3	8.6	4.2	15.2	35.3	55,416
>Secondary school	10.6	13.9	4.5	6.8	2.4	9.3	26.6	4749
Husband’s education								
No education	27.2	24.9	8.8	9.1	5.4	20.6	43.4	48,792
Primary school	23.8	23.6	8.2	9.0	5.2	19.1	41.0	29,649
Secondary school	20.6	22.0	7.3	8.7	4.7	17.5	38.2	84,689
>Secondary school	15.8	18.7	5.5	8.1	3.4	14.0	33.4	11,783
Parity								
1	19.9	19.8	6.7	8.6	4.7	16.0	36.9	53,183
2-3	20.7	22.4	7.3	8.5	4.4	17.6	38.2	74,004
4-5	26.6	26.0	9.1	9.2	5.6	21.4	43.9	29,417
>6	32.1	28.3	10.3	9.8	6.0	24.0	48.2	16,459
Mother’s age (yr)								
<19	23.8	22.9	8.2	9.8	5.0	18.8	41.2	22,416
20-24	21.2	22.2	7.3	8.8	4.7	17.7	38.8	70,023
25-29	22.1	22.8	7.7	8.6	4.8	18.1	39.1	49,488
≥30	25.6	24.3	8.5	8.7	5.4	20.1	42.1	32,986
Mother’s religion								
Hindu	22.6	22.8	7.7	8.8	4.9	18.2	39.7	134,299
Muslims	31.2	29.6	10.0	11.5	6.6	25.8	50.0	22,463
Others	12.5	15.1	5.7	5.8	2.8	10.9	28.1	18,147
Caste								
Scheduled castes	24.7	24.2	8.4	9.4	4.9	19.6	41.6	33,762
Scheduled tribes	16.5	18.2	6.6	7.1	4.2	14.0	32.8	35,195
Other backward castes	24.8	24.1	8.0	8.6	5.0	19.5	41.8	68,996
Forward castes	22.3	23.2	7.5	10.0	5.1	19.2	40.5	33,585
Mother’s employment status								
Unemployed	21.5	22.2	7.4	8.7	4.9	17.4	38.8	97,095
Professional/service/production worker	21.1	23.6	8.7	10.0	5.1	18.4	40.4	10,143
Agricultural worker/farmer/labourer	24.5	23.7	8.1	8.9	4.8	19.9	41.2	67,468
Time and frequency of antenatal care								
No ANC	26.2	24.8	9.0	9.0	6.0	20.3	42.7	52,476
1st visit during first trimester +≥4 visits total	15.3	17.0	4.7	8.0	3.4	13.9	31.5	33,863
1st visit during first trimester +≤4 visits total	22.6	23.4	8.1	8.9	5.0	18.1	40.1	31,282
1st visit during second or third trimester +>4 visits total	17.8	20.8	6.8	9.6	4.1	16.8	36.8	10,503
1st visit during second or third trimester +<4 visits total	25.5	25.2	8.7	9.0	4.9	20.4	43.5	43,652
Place of delivery								
Home	25.5	24.7	8.8	8.9	5.1	20.0	42.4	100,008
Home but attended by an SBA	22.4	22.6	7.9	10.2	5.3	18.6	39.9	9919
Healthcare facility	18.3	20.2	6.2	8.5	4.5	16.0	35.8	64,910
Complications during delivery								
No	13.4	11.1	2.6	3.4	1.7	8.7	23.7	64,861
Yes	28.1	29.8	10.8	12.1	6.8	24.1	49.3	110,052
Region of India								
North	15.9	18.3	5.0	6.9	2.8	14.5	31.9	26,455
Central	29.4	25.4	9.0	9.3	4.8	21.1	44.6	53,687
Northeast	13.5	18.8	5.9	7.6	7.7	13.4	32.6	21,544
East	28.9	30.9	12.1	11.2	7.0	24.0	50.3	42,512
West	17.4	18.8	5.2	8.5	1.8	15.9	34.2	13,976
South	11.8	10.5	1.8	6.3	2.2	10.4	24.5	16,739

ANC, antenatal check-up; SBA, skilled birth attendant.

1 The total population for each variable may vary because of missing observations.

**Table 2.** Proportion of currently married women (aged 15-49 years) residing in rural India during 2007-2008 who sought advice or treatment after experiencing one or more postpartum morbidity within six weeks after delivery

Variables	Sought treatment (%)	Total population^1^		Variables	Sought treatment (%)	Total population^1^
**Individual and household characteristics**				**Community level variables**		
Wealth index				Village connected by an all-weather road		
Poorest	48.3	19,366		No	51.5	11,381
Poor	52.5	18,817		Yes	55.8	50,049
Middle	57.0	15,247		Distance to closest government heslthcare facility (km)		
Wealthy	61.8	11,166		<1	59.8	8,410
Wealthiest	69.4	4,978		1-5	58.1	5,370
Mother’s education				≥5	54.1	55,674
No education	50.9	37,951		Distance to closest private healthcare facility (km)		
Primary school	55.1	10,805		<1	61.0	12,022
Secondary school	62.0	19,562		1-5	57.7	8,264
>Secondary school	71.0	1,263		≥5	53.2	49,160
Husband’s education				Village development index		
No education	50.1	21,172		Poorest	50.1	17,275
Primary school	54.0	12,147		Poor	53.4	15,623
Secondary school	57.5	32,327		Middle	55.2	14,146
>Secondary school	65.3	3,935		Wealthy	57.5	12,835
Parity				Wealthiest	63.1	9,584
1	56.7	19,586		Region		
2-3	55.1	28,224		Central	57.3	8,435
4-5	53.0	12,895		North	54.5	23,930
≥6	53.2	7,931		Northeast	41.7	6,976
Mother’s religion				East	52.3	21,377
Hindu	55.2	53,264		West	65.6	4,774
Muslim	59.5	11,222		South	75.8	4,089
Other	45.5	5,093		**Rural India**	55.1	69,638
Caste						
Scheduled tribes	56.0	14,038				
Scheduled castes	41.4	11,523				
Other backward castes	58.2	28,837				
Forward castes	60.0	13,575				
Mother’s employment status						
Unemployed	56.0	37,655				
Professional/service/production worker	61.5	4,094				
Agricultural worker/farmer/labourer	52.9	27,761				
Time and frequency of antenatal care						
No ANC	42.0	22,380				
1st visit during first trimester + ≥4 visits total	71.8	10,645				
1st visit during first trimester + ≤4 visits total	58.0	12,539				
1st visit during second or third trimester + 4 visits total	65.4	3,859				
1st visit during second or third trimester + <4 visits total	56.6	18,990				
Place of delivery						
Home	48.5	42,354				
Home but attended by an SBA	58.4	3,960				
Healthcare facility	66.3	23,247				

ANC, antenatal check-up; SBA, skilled birth attendant.

1 The total population for each variable may vary if data were missing.

**Table 3.** Unadjusted and adjusted odds ratios (ORs) for treatment-seeking among currently married women (aged 15-49 years) residing in rural India, who experienced one or more postpartum morbidities within six weeks after delivery

Independent variables	Unadjusted		Adjusted	
**Individual and household characteristics**				
Wealth index				
Poorest	1.00		1.00	
Poor	1.18 (1.13-1.23)^0002A;*^		1.03 (0.98-1.07)	
Middle	1.41 (1.35-1.47)^**^		1.08 (1.02-1.13)^*^	
Wealthy	1.71 (1.63-1.80)^**^		1.10 (1.03-1.16)^*^	
Wealthiest	2.40 (2.24-2.56)^**^		1.23 (1.14-1.33)^*^	
Mother’s education				
No education	1.00		1.00	
Primary school	1.18 (1.13-1.23)^**^		1.05 (1.00-1.10)^**^	
Secondary school	1.55 (1.50-1.61)^**^		1.15 (1.10-1.20)^*^	
>Secondary school	2.34 (2.07-2.64)^**^		1.19 (1.03-1.34)^*^	
Husband’s education				
No education	1.00		1.00	
Primary school	1.17 (1.12-1.22)^**^		1.11 (1.05-1.16)^†^	
Secondary school	1.34 (1.29-1.39)^**^		1.06 (1.01-1.11)^**^	
>Secondary school	1.85 (1.73-1.99)^**^		1.12 (1.02-1.21)^*^	
Parity				
1	1.00		1.00	
2-3	0.94 (0.90-0.97)^**^		1.07 (1.02-1.11)^**^	
4-5	0.87 (0.83-0.91)^**^		1.23 (1.17-1.28)^**^	
≥6	0.88 (0.84-0.93)^**^		1.34 (1.28-1.41)^**^	
Religion				
Hindu	1.00		1.00	
Muslims	1.20 (1.15-1.25)^**^		1.30 (1.24-1.36)^**^	
Other religions	0.68 (0.64-0.72)^**^		1.02 (0.93-1.10)	
Caste				
Scheduled tribes	1.00		1.00	
Scheduled castes	0.56 (0.53-0.59)^**^		1.58 (1.52-1.65)^**^	
Other backward castes	1.10 (1.05-1.14)^**^		1.62 (1.56-1.69)^**^	
Forward castes	1.18 (1.12-1.24)^**^		1.52 (1.45-1.59)^**^	
Mother’s employment				
Unemployed	1.00		1.00	
Professional/service/production worker	1.25 (1.17-1.33)^**^		1.17 (1.09-1.25)^**^	
Agricultural worker/farmer/labourer	0.88 (0.85-0.91)^**^		1.05 (1.01-1.09)^*^	
Time and frequency of ANC				
No ANC	1.00		1.00	
1st visit during first trimester + ≥4 visits total	3.47 (3.30-3.64)^**^		2.60 (2.53-2.67)^**^	
1st visit during first trimester + ≤4 visits total	1.89 (1.80-1.97)^**^		1.73 (1.68-1.79)^**^	
1st visit during second or third trimester + >4 visits total	2.57 (2.39-2.76)^**^		2.18 (2.09-2.27)^**^	
1st visit during second or third trimester + <4 visits total	1.79 (1.72-1.86)^**^		1.66 (1.61-1.70)^**^	
Place of delivery				
Home	1.00		1.00	
Home but attended by an SBA	1.49 (1.39-1.59)^**^		1.33 (1.26-1.41)^**^	
Healthcare facility	2.07 (2.00-2.14)^**^		1.59 (1.55-1.64)^**^	
**Community characteristics**				
Village connected by all-weather road				
No	1.00		1.00	
Yes	1.18 (1.13-1.22)^†^		1.00 (0.94-1.06)	
Distance to closest government healthcare facility (km)				
<1	1.00		1.00	
1-5	0.93 (0.87-1.00)		1.00 (0.89-1.10)	
>5	0.79 (0.75-0.83)^*^		0.94 (0.87-1.02)	
Distance to closest private healthcare facility (km)				
<1	1.00		1.00	
1-5	0.88 (0.83-0.93)^*^		0.95 (0.86-1.04)	
>5	0.73 (0.70-0.76)^*^		0.93^*^ (0.86-0.99)	
Village development index				
Poorest	1.00		1.00	
Poor	1.13 (1.08-1.18)^**^		1.00 (0.94-1.07)	
Middle	1.21 (1.16-1.26)^**^		0.99 (0.93-1.06)	
Wealthy	1.32 (1.26-1.39)^**^		1.02 (0.95-1.09)	
Wealthiest	1.67 (1.59-1.76)^**^		1.02 (0.94-1.10)	
Region				
Central	1.00		1.00	
North	0.91 (0.87-0.96)^*^		0.86 (0.79-0.93)^**^	
Northeast	0.55 (0.51-0.58)^*^		0.51 (0.42-0.59)^**^	
East	0.83 (0.79-0.87)^*^		0.88 (0.83-0.94)^**^	
West	1.44 (1.34-1.55)^*^		1.32 (1.22-1.41)^**^	
South	2.36 (2.17-2.57)^*^		1.49 (1.38-1.59)^**^	

Values are presented as OR (95% confidence interval).

ANC, antenatal check-up; SBA, skilled birth attendant.

† p<0.10,

* p<0.05,

** p<0.01.

## Appendix 1. Operational definitions and variable categories used in this study

**Table:**

Variables	Definition and categorisation
**Dependent variable**	
Sought treatment or consultation for a postpartum morbidity (yes or no)	Seeking any treatment for a health problem experienced within six weeks after delivery was coded as ‘0’ and ‘1’ for never or ever seeking treatment or consultation, respectively. Any women who experienced at least one of the following health problems were included: high fever, lower abdominal pain, foul-smelling vaginal discharge, excessive bleeding, convulsions, or severe headaches
**Independent variables**	
Individual and household characteristics	
Wealth index	A composite index of the household amenities and assets that were divided into quintiles as ‘1’ (lowest income), ‘2’, ‘3’, ‘4’, and ‘5’ (highest income)
Parity of the mother	The number of total children a mother had given birth to at the time of the survey
Husband’s education level	The maximum number of years of schooling a husband received. This variable was coded as ‘0’ for no schooling, ‘1’ for up to 5 years of schooling (primary school), ‘2’ for 6 to 9 years of schooling (secondary school), and ‘3’ for 10 or more years of schooling (above secondary schooling)
Mother’s education	The maximum number of years the mother attended school was coded as ‘0’ for no schooling, ‘1’ for up to 5 years of schooling (primary school), ‘2’ for 6 to 9 years of schooling (secondary school), and ‘3’ for 10 or more years of schooling (above secondary schooling)
Religion	The mother’s religion was coded as ‘0’ for Hindu, ‘1’ for Muslim, and ‘2’ for other religions, which included Sikhism, Christianity, Jainism, Buddhism, Zoroastrianism, and other faiths/tribal religions
Caste	Mother’s caste was coded as ‘0’ for the Scheduled Castes, ‘1’ for the Scheduled Tribes, ‘2’ for the Other Backward Castes, and ‘3’ for the Forward Castes. These are the official categories used by the Government of India. The social system in India is characterised by numerous castes irrespective of religion. The castes that were/are deemed elite by the Indian society are classified as the “general category” or in the case of this study as the Forward Castes. The other communities, which have been socio-economically backward in the past, are categorized as ‘lower castes’. These lower castes are further divided based on their status in the society as Other Backward Classes and Scheduled Castes. Scheduled Castes comprise groups that were previously considered ‘untouchables’. The category of Other Backward Classes comprises castes other than the elites and untouchables and is economically and socially backward. Scheduled Tribes are those groups or tribes that did not practice the caste system and lived in hilly, forested, and remote areas secluded from the mainstream Indian society. The Scheduled Tribes and Scheduled Castes comprise approximately 7% and 16% of total population of India, respectively. The population of the Other Backward Classes varies according to the source, but was estimated to be as high as 52% of the total population according to the 1980 report of the Second Backward Classes Commission by the Government of India. The general category or the Forward Castes comprise approximately 25% of the total population of India
Mother’s employment status	The mother’s employment status during the previous 12 months from the date of interview was coded as ‘1’ for unemployed mothers, ‘2’ for mothers working in the professional/service/production industries, and ‘3’ for mothers working as agricultural workers/farmers/labourers. Unemployed mothers were defined as any mother who stayed at home and did not work outside the home
Antenatal check-up (ANC) timing and frequency	The time of the first ANC and the frequency of all ANCs during the pregnancy were coded as ‘0’ for mothers who did not receive an ANC, ‘1’ for mothers who had their first antenatal check-up (ANC) in the first trimester and also had four or more ANCs in total, ‘2’ for mothers who has their first ANC in the first trimester but had less than four ANCs in total, ‘3’ for those mothers who has their first ANC in the second or third trimester and had four or more ANCs in total, and ‘4’ for those who had their first ANC in the second or third trimester but had less than four ANCs in total
Place of delivery and skilled birth attendance	The type of location where the delivery occurred and whether the delivery was attended by a skilled healthcare provider/professional (doctor, nurse, lady health visitor and auxiliary nurse, or mid-wife) were coded as ‘1’ for delivery at the home not attended by a skilled healthcare provider, ‘2’ for delivery at the home and attended by a skilled healthcare provider, or ‘3’ for delivery in a healthcare facility
Community-level variables^1^	
Village connected by all-weather road	Officials were asked whether the village was accessible by an all-weather road. If yes, then the variable was coded as ‘1’, and if no, then the variable was coded as ‘0’.
Distance to the closest government healthcare facility	Distance to the nearest government healthcare facility such as a health sub-centre, primary health centre, or community health centre were coded as ‘1’ if the facility was within one kilometre from the village or was within the village, ‘2’ if the facility was one to five kilometres from the village, and ‘3’ if the facility was more than five kilometres from the village. This distance was based on self-reported data, thus may not accurately represent the distance between the village and the healthcare facility.
Distance to a private healthcare facility	The distance to the nearest private healthcare facility including clinics, nursing homes, and hospitals were coded as ‘1’ if the facility was located within one kilometre from the village or was within the village, ‘2’ if the facility was one to five kilometres from the village, and ‘3’ if the facility was located more than five kilometres form the village. This distance was based on self-reported data, thus may not accurately represent the distance between the village and the healthcare facility
Village development index	The village index was based on the available facilities such as banks, post offices, medical shops, youth clubs, markets, a telephone booth, and others. The index was calculated using a principal component analysis using Cronbach’s alpha values (Appendix 2). The index was divided into quintiles from poorest to wealthiest (1-5)
Regions	The regions of the states in Indian were determined based on the second National Family Health Survey (1=north region and included Jammu and Kashmir, Himachal Pradesh, Punjab, Rajasthan, Haryana, Chandigarh (the Union Territory) and Delhi; 2=central region and included the states of Uttar Pradesh, Uttaranchal, Madhya Pradesh and Chhattisgarh; 3=northeast region and included the states of Sikkim, Assam, Meghalaya, Manipur, Mizoram, Nagaland, Tripura, and Arunachal Pradesh; 4=east region and included the states of Bihar, Jharkhand, West Bengal, and Orissa; 5=west region and included the states of Gujarat, Maharashtra, Goa, the Union Territory of Dadara and Nagar Haveli, and Daman and Diu, 6=south region and included the states of Kerala, Karnataka, Andhra Pradesh, Tamil Nadu, and the Union Territories of the Andaman and Nicobar Islands, Pondicherry, and Lakshadweep)

1 All community-level variables, except region, were constructed using the village data file. Information about the village was gathered from officials in the village such as the pradhan/up-pradhan (Head/Deputy-Head of the village), any other panchayat (village committee) members, teachers, Gram Sevak (village government workers), and Anganwadi workers (local health workers).

## Appendix 2. Cronbach’s alpha values for the village development index

**Table:**

Variables	α-value	Variables	α-value
Drainage facility present	0.79	Adult education centre present	0.79
No electricity available	0.79	Youth club present	0.79
Electricity supplied <6 hr	0.80	Women’s club present	0.79
Electricity supplied >6 hr	0.78	Self-help groups available	0.79
Village <5 km from the nearest town	0.79	Haat/Market available	0.79
Village 5-10 km from the nearest town	0.80	Credit cooperative society	0.78
Village 10-20 km from the nearest town	0.80	Agricultural cooperative society	0.78
Village >30 km from the nearest town	0.79	Milk cooperative society	0.79
Post office present	0.78	Fishermen’s cooperative society	0.80
Telephone booth present	0.78	Public computers/*e-chaupal* present	0.79
Pharmacy/medical supply shop present	0.78	Mills/small scale industries present	0.79
Bank present	0.78	Community television available	0.79
**Test scale**			**0.80**
